# Mapping Information Needs of Patients With Sexually Transmitted Infections Using Web-Based Data Sources: Grounded Theory Investigation

**DOI:** 10.2196/30125

**Published:** 2021-11-10

**Authors:** Pavankumar Mulgund, Raj Sharman, Sandeep Purao, Sagarika Suresh Thimmanayakanapalya, Peter Winkelstein

**Affiliations:** 1 Department of Management Science and Systems State University of New York at Buffalo Buffalo, NY United States; 2 Department of Information & Process Management Bentley University Waltham, MA United States; 3 Department of Pediatrics, Jacobs School of Medicine and Biomedical Sciences State University of New York at Buffalo Buffalo, NY United States

**Keywords:** information needs, sexually transmitted diseases, patient journey maps, health information seeking, stigmatizing disorders, online forum, sexually transmitted infection, American Sexual Health Association, grounded theory, stigma

## Abstract

**Background:**

According to the World health organization (WHO), more than 1 million sexually transmitted infections (STIs) are acquired each day across the world. The incidence rates of STIs in the United States are at a record high for the fourth consecutive year. Owing to the stigma associated with the incidence of STI, there is a general reluctance to seek information in person. Instead, web-based information sources remain the primary avenues of information-seeking. However, these sources are designed without a comprehensive understanding of the information needs of individuals who have contracted STIs.

**Objective:**

This study aimed to investigate the information needs of individuals who have or suspect they have contracted an STI. A better understanding of their information needs can drive the design of more effective digital interventions.

**Methods:**

This is a qualitative and analytical study of 549 transcripts (consisting of queries posted over the last 10 years) from web-based forums of the American Sexual Health Association (ASHA), which allows patients, volunteers, and health care providers connect anonymously. The analysis follows a grounded theory (GT) approach with multiple coding stages to uncover categories and themes.

**Results:**

Three categories of information needs emerged. The first two, *clinical* and *logistical*, are similar to other contexts. However, our analysis shows that there is a significant need for the last category—*psychosocial* information. Approximately 59% of instances are linked to concerns such as confusion, discretion, remorse, and others. These needs vary across the stages of a patient’s journey from symptom manifestation to treatment maintenance.

**Conclusions:**

Responding to the needs of individuals who have or suspect they have contracted an STI requires compassionate and personalized responses (beyond factual clinical and logistical information). Web-based forums provide anonymity but do not adequately incorporate mechanisms, practices, or incentives to respond to diverse psychosocial concerns. Innovative approaches to add such support can make the digital interventions more effective for this group of individuals.

## Introduction

### Background

The incidence of sexually transmitted infections (STIs) remains high across the globe and within the United States [1,2]. According to a report released in January 2021, the US Centers for Disease Control and Prevention estimated that on any given day in 2018, one in 5 people in the United States had an STI [3]. Consequences include acquiring HIV, mother-to-child transmission resulting in stillbirth, congenital deformities, and other complications such as cervical cancer [4]. Within the United States, the annual cost of treating and managing STIs is estimated to be more than US $16 billion [5].

Despite this scale and such severe consequences, many individuals who have or suspect they have contracted an STI do not readily consult their physician. Owing to the stigma associated with the incidence of STIs, their primary source of information appears to be web-based forums, where they can anonymously seek information from other patients, volunteers, and health care providers [6]. These forums facilitate questions and answers while protecting the identity of individuals. However, individual contributors and designers of these forums do not appear to possess an understanding of the information needs [7] of this population.

In this paper, we developed an in-depth understanding of the information needs of individuals who have or suspect they have contracted an STI following a grounded theory (GT) approach. The analysis relies on secondary data (queries posted) on a web-based forum. The results are described as categories of information needs associated with stages of the patient journey. The results are important because they inform our understanding of how individuals search for information about taboo-ridden disorders; and they are useful because they can inform the design of digital interventions in response.

### Rationale

Information-seeking describes an individual’s conscious effort to acquire information in response to a need or gap [8]. An information need emerges from the realization that one’s knowledge is insufficient to satisfy a goal at hand and then culminates in locating information that contributes to understanding and meaning [9]. Information-seeking is a complex phenomenon characterized by the interplay of seekers’ personal needs, learning styles, available resources, and affective components, among others [10]. It begins when one realizes the need for information and ends when that need is believed to have been satisfied [11].

Health information–seeking has been defined as a “problem-focused coping strategy … adopted by individuals as a response to a threatening situation” [12], which may also include “the urge to confront oneself with the threatening situation by means of seeking more information about it” [13]. The problem is important because health care providers as well as researchers are interested in understanding “how and why individuals obtain health information, where they go to retrieve such information, what particular types of information they prefer, and how the health information sought is used” [14].

Much prior scholarship points out that information needs can vary on the basis of the disease or condition. In addition to clinical information like symptoms and treatments; individuals may also seek emotional support, anonymity, and privacy, especially for conditions (such as STIs) whose incidence invokes stigma [15,16]. In these cases, individuals answering the questions (physicians) may also prefer anonymity. Researchers have attempted several innovative techniques to encourage a more open dialogue with patients with STIs about their information needs. In addition to the traditional methods such as focus groups and structured interviews, novel techniques such as concept cards [17], photo-based projective techniques [18], computer-assisted video elicitation [19], and vignettes [20] have been used. However, the success of these methods is associated with the facilitator’s skills and can generate incomplete or incorrect outcomes [21]. Consequently, eliciting and understanding the information needs of a patient with an STI remains a challenging endeavor. This is the precise challenge we undertake in this study.

## Methods

### Methods Overview

We used a GT approach relying on secondary data analysis [22]. Use of secondary data sources in GT approaches is quite common. Following the idea that all is data, grounded theorists have used a variety of sources such as documents [23], text, and transcripts of written material [24,25]. However, 2 issues assume importance when conducting a GT approach using secondary data. First, the secondary data source should be rich in terms of volume and diversity to develop a generalizable theoretical framework. Second, the researchers should use contextual positioning [26] to collect the data for analysis, which involves approaching data reflectively to establish the context. In this study, we address both these issues, as described in the following sections.

We adopted a GT approach because it is appropriate for deciphering shades of meaning, implied pointers, and included efforts to traverse between an utterance or a phrase or sentence to the larger context of the discourse. We considered the possibility of automated analyses, which offers scale and efficiency, but decided not to pursue it because, in its current level of maturity, automated analysis may not produce as conceptually rich observations as GT. Such methods also require significantly larger data sets and may lead to spurious correlations at lower volumes of data.

As a descriptive approach that emphasizes the discovery of central concepts, GT is well suited to uncover the information needs of patients with STIs. GT is also a good fit because of a lack of prior theoretical work that explains the information needs of individuals who are affected by a taboo-ridden condition such as an STI. Although much prior work [27,28] has discussed patients’ information needs at a more general level, it has not accounted for the unique challenges that the stigmatizing disorders (such as STIs) present. When applied rigorously, GT is a robust methodology that fits well in practical situations with a real-world orientation. It is an iterative approach, where researchers interlace data collection and analysis until “concepts” emerge. The concepts are interpreted in response to the research goal, which in this case is to understand the information needs of individuals who have or suspect they have contracted an STI.

As patterns begin to emerge, the concepts are classified and tied to the context, which in this case is patient journeys, grounded in the realities of seeking information when faced with different uncertainties. We applied the GT approach with the following specific decisions.

### Data Collection

We collected transcripts of questions and responses from the Question-and-Answer forum of the American Sexual Health Association (ASHA), a nonprofit organization operating in the United States. In line with its mission to “empower individuals, families, and communities to achieve sexually healthy lives through education and advocacy,” ASHA offers a web-based community that connects patients, families, friends, and caregivers to support individuals with STIs. The web-based portal sponsored by ASHA and maintained by the Inspire community allows individuals who have or suspect they have contracted an STI to ask STI-related questions. The volunteers who respond to questions include caregivers (nurses and physicians), social workers, health communication experts, and individuals with prior experience managing their STIs. Although the content can be generated by any member participating in the community, the threads are moderated by experts. The portal requests nonidentifiable information from the contributors, such as age, gender, and location.

**Figure 1:**
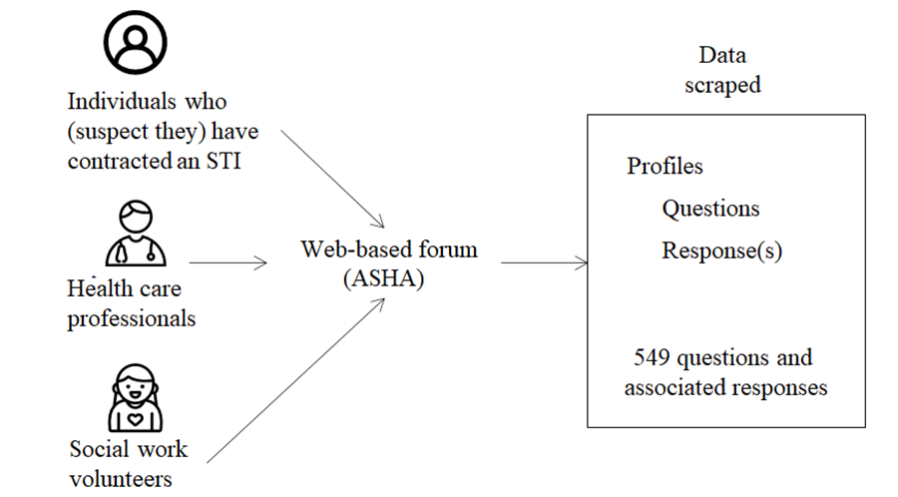
Data collection from the web-based forum. ASHA: American Sexual Health Association, STI: sexually transmitted infection.

After cleansing the data, we obtained 549 transcripts, which comprised questions and associated responses. For each transcript we collected (1) the query posted by the individual who has or suspects he/she has contracted an STI, (2) responses to the query posted by other participants on the portal, and (3) nonidentifiable information of each contributor on their profile (Figure 1).

### Data Analysis

**Figure 2:**
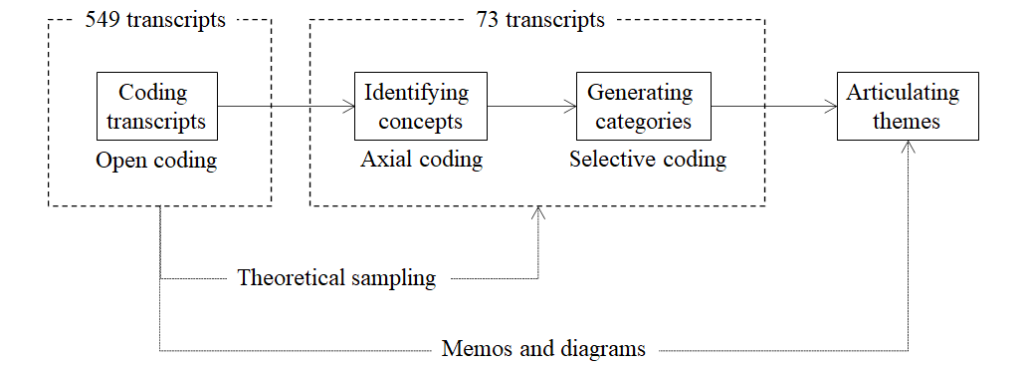
Data analysis across 3 phases.

Data analysis included generating summaries of participant demographics, followed by a characterization of questions for each demographic, and finally, an analysis of the content of the transcripts. During this content analysis, the first phase involved analyzing the complete set (549 transcripts) sequentially with open coding (extraction of keywords and phrases, and assigning codes to each [29]), which resulted in 115 unique codes. The results were examined to identify a subset (73 transcripts) on the basis of the uniqueness of codes they generated. This selection was thus nonprobabilistic, and followed theoretical sampling guidelines stipulated by prior research [30]. The second phase of the analysis (with 73 transcripts) involved revisiting these transcripts for more intense analysis with the “constant comparative method” [29]. We revisited the transcripts to compare codes extracted from the initial transcripts against those from later transcripts. This phase also involved axial coding (grouping the codes to identify code clusters [29]), which resulted in 43 categories. The last phase of the analysis process involved selective coding, which included identifying and articulating higher-level themes [29]. Theoretical saturation was observed in this iterative process as the number of new codes dropped to near-zero. Across these phases, we generated several memos and representations to record details, and capture different interpretations. The effort was managed with the Atlas.ti software. Figure 2 outlines the data analysis process.

Table 1 shows an example of outcomes from the coding process to illustrate the path from raw data to the discovery of concepts. The effort was data-driven but influenced by the authors’ exposure to prior research [31].

An important ancillary outcome from our analysis was the recognition that the individual patients appeared to follow a journey that started with symptom manifestation and progressed through several stages to ongoing maintenance of the disease.

**Table 1:** An example of data coding.

Raw data	Phase 1: open coding	Phase 2: axial coding	Phase 3: selective coding
“I just found out today that I tested positive and I am lost for words I can't stop crying I don't know what to do I've been married for 3 years made the biggest mistake of my life went out my marriage now I don't know what to do or how to tell my partner”	Feeling miserable Feeling anxious Feeling lost Feeling remorseful	Managing negative sentiments	Psychosocial information needs
I'm driving myself crazy on google.”	Struggling with lack of relevant information Overwhelmed by internet-based content	Incomplete information Concern about misinformation	
“The thought of even meeting a man and having to explain it all over again to a new person never feels good*.*”	Fear of disclosing information Worrying about not being able to have a child	Worries about future relationships Anxiety about bleak prospects	

## Results

### Participant Characteristics

The participants in the forum, 87 individuals (47 women, 29 men, and 11 not specified) ranged in age from 18 to 74 years (women: 20 to 52 years, median ~29 years, average ~30 years, mode ~34 years; men: 18 to 74 years, median ~43 years, average ~42 years, mode ~49 years). As many as 2 out of 3 queries were posed by individuals aged between 18 and 35 years. Only 5 individuals described themselves as part of the LGBT (lesbian, gay, bisexual, and transgender) community (self-reported, does not necessarily suggest lack of participation from the most vulnerable population segment). The data do not include individuals who may have visited the forum to read posts from others (without asking or responding to a question). Despite these caveats, 62% of the posted questions were from female information seekers. Most frequent infections discussed were herpes, syphilis, chlamydia, genital warts, and gonorrhea (a total of 503 occurrences). Very few (3 occurrences) of preventive behaviors discussed and included condom use and vaccinations.

### Categories of Information Needs

**Figure 3:**
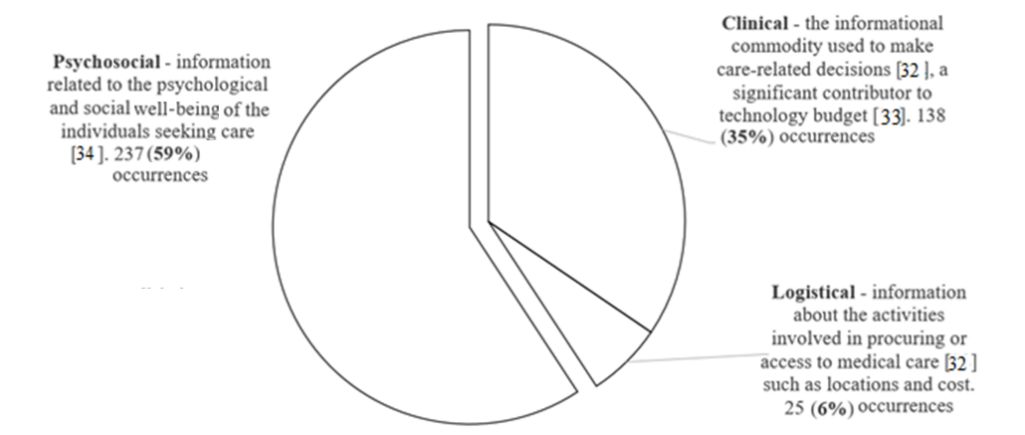
Categories of information needs.

The key finding was the different categories of information needs. Here, the unexpected finding was that as many as 59% of the queries belonged to the category of *psychosocial information needs*, indicating a set of diverse concerns. Figure 3 summarizes these findings.

#### Category 1: Clinical Information Needs

The first category, *clinical information needs* is defined as the informational commodity used to make care-related decisions [32] and accounts for a significant part of information technology budgets within the health care organizations [33]. It included details about the conditions, symptoms, risk factors, epidemiology (how the disease spreads), possible differential diagnosis, clinical presentation, and prognosis. The following excerpt illustrates this further.

I had unprotected sex with a man last week and afterwards, he told me that his previous partner has HSV-2. He told me that she hasn’t had an outbreak in years and that he’s never had an outbreak. Should I be concerned about contracting HSV-2? I was going to get tested in a month and see what the results are. Any information will help.

The clinical information sought may include diagnosis and initial treatment and other relevant information such as treatment costs and access. The following excerpt illustrates this further.

If I’ve catched chlamydia conjunctivitis only on my left eye as a first contact with the germ, for example from touching with my hand a infected fluids and then with the same hand touching my eye (No penetration, or oral sex). Is it possible the chlamydia goes to my sexual organs with the pass of time? (Considering that the first contact with the germ was in the eye).

The individual seeking such information often shared personal circumstances in an effort to seek specific clinical information and even offered advice to others. The following excerpt illustrates this further.

Today I begin the transition to a new apartment as my disease has progressed to the point of not being able to climb stairs anymore. I can barely walk on level ground as the bacteria that has been living in my body for apparently many, many years has irreversibly damaged my nervous system. If you have been sexually active in the past 20 years, please get tested for Syphilis before it gets to the “tertiary” stage and you wind up disabled like me.

#### Category 2: Logistical Information Needs

The second category, *logistical information needs* is defined as information about activities involved in procuring and access to medical care [32]. It included 2 broad subcategories. The first was *financial considerations* such as insurance coverage or out-of-the-pocket cost of medication and treatment. The following excerpt illustrates this further.

I've been advised by 3 doctors that I NEED surgery for the removal of anal warts and most likely are internal, anyone been through the surgery in the anal area? What’s the cost like? process? HELP ME I am so scared, I've been infected since December of 2010 I've had a few freezing treatments, it never worked for me, now in need of surgery.

The second subcategory was information about providers, testing facilities, appointment timelines, and reviews about providers and test efficacy. Questions about educational resources, contraceptives, and helplines were also discussed. The following excerpt illustrates this further.

I am unable to find information on any website with a suggested timetable from exposure for STD testing. Does anyone know this information/suggestion where to look? Which diseases should be retested in another period of time?

#### Category 3: Psychosocial Information Needs

The third category, *psychosocial information needs* is defined as the information related to the psychological and social well-being of the individuals seeking care [34]. These needs were not only the most complex but also the most idiosyncratic [35,36]; they are also highly sought after and least well-addressed, as evidenced by the appearance of high-sentiment words such as “shocked,” “worried,” “depressed,” “scared,” and “fear” in 82% of the transcripts. Psychosocial needs emerge to cope with the negative emotions involved with a general feeling of not being healthy [37]. Such needs are amplified in stigmatizing topics such as those relating to STIs. “Psychosocial needs” vary considerably through each phase of the patient's journey. Several excerpts illustrate this further.

I had unprotected sex 3 days ago and suddenly have a tingling/pain feeling in my inner thig/groin and kind of throughout my leg. I am kind of freaking out because I'm thinking the worse, i might just be paranoid can you please help me figure out what this is. No other symptoms just what i described above.

The concerns expressed by participants included the shock of becoming infected with STIs, the despair and anger associated with the outcome.

I feel like my world is shattered. I’ve cried an ocean and am still trying to process all this. I’m just looking for a support group on how to cope with this

Participants described the feeling of being dirty or contaminated with extreme negative emotions, including suicidal tendencies, as highlighted in the transcripts below.

I just started dating another woman about 2 months ago when I noticed a small bump... like a pimple... on my penis. I went to my doctor thinking it was a benign skin tag. I had skin tags removed from other parts of my body in years past. I was shocked... absolutely shocked when the doc told me that the bump was a genital wart. I feel dirty, trashy and disgusted.

Some worried about being judged by the providers and talked about embarrassments because of disclosing unpleasant and discrediting details about prior sexual behavior. The following transcripts illustrate these issues.

I have seen multiple doctors and most have said they look like very tiny warts (almost microscopic) but are too small to treat since any of the treatments used would ruin healthy surrounding skin. Two docs even said not worry since “I look like a healthy person” and they should clear on their own. That was 15 years ago. Some docs think its from another type of genital hpv other than 6 or 11. This is so frustrating and I am now in my 40s. I have seen over 8 doctors.

Two other issues were also frequently discussed. These included worries about treatment adherence with complex treatment regimens and fear of dealing with associated pain.

I know it’s not the end of the world and it’ll get better, but it’s just fresh and the pain in HORRIBLE, anyone have advice?? Or remedies to make the outbreaks less pain.

Concerns about revealing to current and prospective partners is another common concern. Although there are regulations for partner communication and contact-tracing, there is inadequate guidance about on how to (1) engage in such difficult conversation and (2) deal with the anxiety of revealing such information to a partners.

These concerns are especially pronounced among middle-aged and senior information seekers.

The thought of even meeting a man and having to explain it all over again to a new person never feels good. The thought of being single after not being single for so long is anxiety-provoking enough.

There was also worry about postincidence sex life and the fear of passing the infection to others. This was amplified among participants who feared the negative consequences to their reproductive health.

I was hoping to find other moms who have breastfeed their children successfully with HSV-2. I have been exclusively pumping and bottle feeding due to an article I read about contracting HSV through breastfeeding and so I’ve stopped but desperately want to breastfeed and was hoping I’m being completely irrational about this. I’ve never had an outbreak on my nipples nor did I realize that was possible. Hoping to be able to breastfeed again without risking my baby’s health.

### Patient Journey

The ancillary finding from the analysis—the patient journey—also revealed how individuals in this group progressed through different stages. We observed several references about patient stages (temporal progression of events, feelings, and things) in the question transcripts during the coding and memoing phases. On mapping the emergent codes to patient stages, we arrived at a tentative flow of patients seeking information on STIs. Subsequent literature review revealed the concept of patient journey maps and its fit with the goal of this study. Therefore, drawing on ideas from patient journey maps [38], we developed a conceptualization of how individuals progress through different stages of experience [39-41].

Adopting a clinical focus, prior scholarship has also created disease progression flows highlighting changes in signs and symptoms over time [42]. Although there is some alignment between patient journey and disease progression, patient journey map is a broader concept and presents the various stages of patient experience, including touchpoints with care providers, and feelings and emotions they experience. We present the details below.

**Figure 4:**
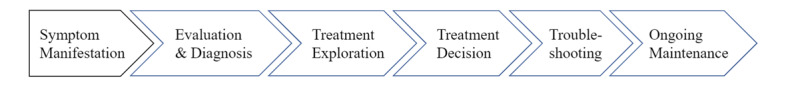
Patient journey.

The journey begins when an individual experiences symptoms, which may lead to evaluative tests and diagnosis, and an exploration of treatment options before reaching a treatment decision. Follow-ups for any troubleshooting would eventually lead ongoing maintenance of the disease. These stages were identified by examining the codes and synthesizing the ideas across transcripts. Figure 4 summarizes the patient journey.

The journey map concept has been used in prior research to explore needs across different stages in case of patients with cancer [43,44] or patients undergoing surgical interventions [45]. With the help of these ideas, we were able to interpret our findings; for example, by mapping the types of questions against different stages of the patient journey (Figure 3). Specifically, we found that the clinical information and psychosocial information needs varied considerably across the stages of the patient’s journey map, whereas logistical information needs did not.

### Information Needs Across the Patient Journey

**Figure 5:**
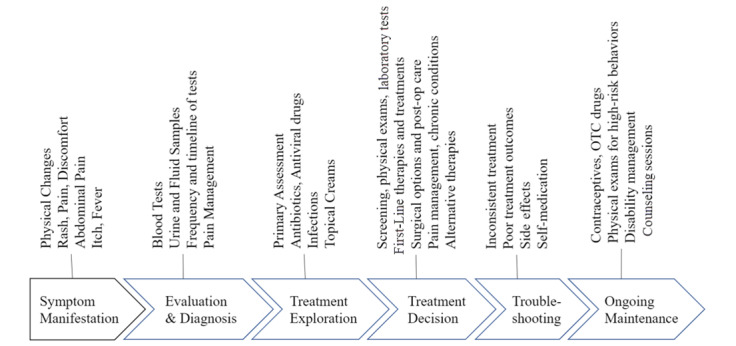
Clinical information needs during the patient journey. OTC: over-the-counter.

The specific types of information needs for each category varied across the stages of the patient journey. For example, clinical information needs in the evaluation and diagnosis stage were about tests, samples, and pain management; these changed to treatment outcomes and side-effects during the troubleshooting stage. Figure 5 summarizes these needs.

**Figure 6:**
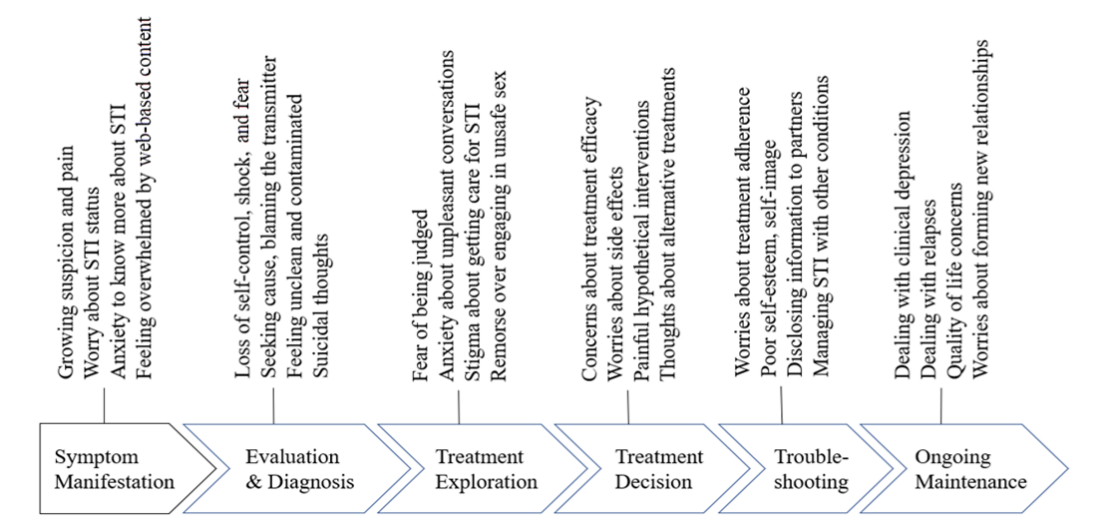
Psychosocial information needs during the patient journey. STI: sexually transmitted infection.

The psychosocial information needs during the evaluation and diagnosis stage were about loss of self-control, seeking cause and blaming, and feeling contaminated; these transitioned to worries about treatment adherence, poor self-image, and disclosing information to partners during the troubleshooting phase. Figure 6 summarizes these needs.

The last category of information needs (logistical information) was not analyzed in this manner because of the small number of instances. Relative frequencies of the two categories (clinical and psychosocial information needs) were computed for each stage. Table 2 summarizes this information.

**Table 2:** Relative frequencies of information needs across stages of the patient journey.

Journey stage	Symptom manifestation, %	Evaluation and diagnosis, %	Initial treatment exploration, %	Treatment decision, %	Troubleshooting, %	Ongoing maintenance, %
Clinical needs	36.22	16.69	21.86	8.32	12.66	4.23
Psychosocial needs	31.50	18.32	12.46	11.44	19.20	7.12

## Discussion

### Principal Findings

This research adds to a stream of work that has explored information needs and information-seeking in general [46,47] and for specific populations [48]. Some of these studies have explored the information needs of women with HPV [49], health information needs of ethnically diverse adolescent patients with STIs [50], and preventive information needs among HIV-negative individuals [51]. Our work is unique in that it provides a comprehensive depiction of the information needs and includes three categories, *clinical*, *logistical*, and *psychosocial*, which are mapped across each stage of the patient journey.

A key finding is a complex set of psychosocial information needs, some of which vary with each stage of the patient journey and others that persist across stages. More importantly, we found that psychosocial information needs are most critical for individuals who have or suspect they have contracted an STI. This category of information needs remains least explored in prior work and is likely to present significant barriers to information access, further exacerbated by patients’ general reluctance to seek in-person help and the lack of information sources that cater to these needs. Unlike clinical and logistical information needs that are well catered to by current information sources, there appears to be a lack of appropriate or tailored sources that can respond to the psychosocial information needs. Additionally, the study also points to a lack of emphasis on information-seeking for preventive behaviors.

### Implications for Practice

This work has significant implications for practice by informing the design and development of information sources. Current information sources (general and specialized search engines and information portals) can respond to the patients’ clinical and logistical information needs but do not consider their psychosocial information needs. Our findings highlight their relative importance of this category of information needs (approximately 59%) from a patient’s perspective and can therefore encourage future designers to develop information sources as well as discussion forums that respond to this deficiency. Our findings are timely as we witness a surge in the number of people seeking information on the internet.

From an information-seeking perspective, our study underscores the need to cater to the emotional challenges of the individuals who have or suspect they have contracted an STI. Broadly, our study alludes to the need to understand and explore the information needs of individuals struggling with stigmatizing conditions. More research is warranted to explore similarities and differences among such disorders, and how we can respond to the information needs from these populations.

### Limitations

There are limitations to this work. First, the study relies on data gathered from ASHA—one of the largest forums for patients with STIs in the United States. Even though ASHA contains a large volume of questions and responses, it may not reflect all of the information needs of patients with STIs. Analyzing information from other websites may provide additional insights. Further, ASHA caters mostly to people living in the United States. Therefore, the results may not entirely reflect the differences in the information needs of individuals in other settings.

Second, although the study uncovered the *information needs* for individuals in this group, it cannot fully describe their *information-seeking behaviors*. Third, this work consolidates the patient journey into a single representation. Although it is grounded in rigorous and systematic mapping, the patient journey map is a generalized pathway, and individual patients may not always conform. The findings of this study may not apply to such nonconforming patients. Furthermore, this work does not account for variation among different STIs. Future work can examine additional sources, explore information-seeking behaviors, and capture pathways for subgroups of patients. Lastly, although it is unlikely that information portals (such as WebMD) instead of and web-based forums (such as ASHA) can address all the information needs of individuals from this group [50], it is important to explore how individuals may combine these sources.

### Conclusions

The findings of this study emphasize that the information needs of individuals who have or suspect they have contracted an STI includes a range of information, including logistical and psychosocial queries, and not just clinical information. Psychosocial information, which is diverse and idiosyncratic, is sought most frequently. These information needs vary across the patient journey stages. The findings have significant practical implications for organizations providing web-based medical information and designing forums that allow patients, volunteers, and providers to connect. The incidence of an STI represents an example of an outset of a stigmatizing medical condition that prevents open information-seeking. This work highlights several specific components of information needs and some peculiar problems that are associated with such stigma. Based on the classification of information needs and its mapping to the patient journey, future work can contribute to the design of more compassionate and personalized responses to information needs with emerging technologies such as conversational AI and others.

## Abbreviations

ASHA: American Sexual Health Association
GT: grounded theory
LGBT: lesbian, gay, bisexual, and transgender
STI: sexually transmitted infection
